# sRNATarBase 3.0: an updated database for sRNA-target interactions in bacteria

**DOI:** 10.1093/nar/gkv1127

**Published:** 2015-10-25

**Authors:** Jiang Wang, Tao Liu, Bo Zhao, Qixuan Lu, Zheng Wang, Yuan Cao, Wuju Li

**Affiliations:** 1 Center of Computational Biology, Beijing Institute of Basic Medical Sciences, Haidian district, Beijing 100850, China; 2 Department of Laboratory Medicine, Jinan Military General Hospital, Jinan, Shandong 250031, China

## Abstract

Bacterial sRNAs are a class of small regulatory RNAs of about 40–500 nt in length; they play multiple biological roles through binding to their target mRNAs or proteins. Therefore, elucidating sRNA targets is very important. However, only targets of a few sRNAs have been described. To facilitate sRNA functional studies such as developing sRNA target prediction models, we updated the sRNATarBase database, which was initially developed in 2010. The new version (recently moved to http://ccb1.bmi.ac.cn/srnatarbase/) contains 771 sRNA-target entries manually collected from 213 papers, and 23 290 and 11 750 predicted targets from sRNATarget and sTarPicker, respectively. Among the 771 entries, 475 and 17 were involved in validated sRNA–mRNA and sRNA–protein interactions, respectively, while 279 had no reported interactions. We also presented detailed information for 316 binding regions of sRNA-target mRNA interactions and related mutation experiments, as well as new features, including NCBI sequence viewer, sRNA regulatory network, target prediction-based GO and pathway annotations, and error report system. The new version provides a comprehensive annotation of validated sRNA-target interactions, and will be a useful resource for bacterial sRNA studies.

## INTRODUCTION

Bacterial sRNAs are a class of small regulatory RNAs of about 40–500 nt in length (1). They comprise three groups (2). The first group includes cis-acting sRNAs, which perfectly match their targets with overlapping regions. The second group is comprised of trans-acting sRNAs, which imperfectly match their targets and show separation regions. The third group includes protein-binding sRNAs. Since the targets of cis-acting sRNAs are relatively easy to identify, we mainly focused on the latter two groups, especially the second group. sRNAs play important roles in many biological processes through binding to their target mRNAs or proteins (3). For example, five sRNAs (IsrJ, IsrM, IstM, OxyS and SroA) are involved in *S*. *typhimurium* virulence via interaction with target mRNAs such as hilE and sopA (4). Additionally, the interaction of CsrA and CsrB in *E.coli* regulates glycogen production (5,6): CsrB is a sRNA of 360 nucleotides in length, while CsrA is an RNA-binding protein of 61-amino-acids. CsrA can repress glycogen synthesis by binding and destabilizing glgCAP or other mRNAs. However, overexpression of the *E.coli* CsrB gene results in CsrA^−^ phenotype, indicating that this sRNA antagonizes CsrA effects on glycogen synthesis. Therefore, identifying sRNA targets is very important in sRNA functional studies.

To date, multiple experimental and computational methods have been developed for the identification of sRNAs and their targets (3,7,8), with related databases designed, including RegulonDB (9), sRNAMap (1), sRNAdb (10), Rfam (11), BSRD (12) and NPInter v2.0 (13). RegulonDB is a comprehensive database for transcriptional regulation in *E.coli*, and contains 110 sRNAs and 227 sRNA-target interactions. Among these interactions, 53 binding regions of target mRNAs are known and 50 sRNAs are involved, but with no detailed information regarding the binding regions of sRNAs and related mutation experiments. sRNAMap includes 908 sRNAs from gram-negative bacteria. However, this database only provides 57 sRNA targets without information regarding binding regions. sRNAdb is an sRNAs database for gram-positive bacteria, which includes 671 validated and 9993 prediction-based sRNAs, with no sRNA-target interactions provided. Additionally, Rfam is a database of functional RNA families, each of which is represented by a multiple sequence alignment and a covariance model. A total of 3115 bacterial sRNAs are included in this database, but no information regarding sRNA-target interactions is available. The comprehensive sRNA database BSRD contains 897 validated sRNAs and 203 sRNA-target interactions, some of which were derived from sRNATarBase 2.0 (2). However, no binding regions of sRNA-target mRNAs interactions were provided. A total of 57 sRNAs are involved in these interactions. Finally, NPInter v2.0 mainly provides interactions between non-coding RNAs and other biomolecules in *Homo sapiens* and *Mus musculus*. There are only 107 bacterial sRNA-target interactions from four organisms, including *B. subtilis*, *E.coli*, *S. typhimurium* and *S. aureus*. Among them, 32 sRNAs are involved, but no binding regions are provided. In summary, the aforementioned six databases do not provide comprehensive information about sRNA-target interactions, their binding regions and related mutation experiments. Therefore, they cannot be applied to developing prediction models of binding regions of sRNA–mRNA interactions. Additionally, only targets of a few sRNAs have been described. Moreover, the application of high-throughput sequencing in sRNA discovery has resulted in increasing number of reported sRNAs. Thus, the targets of a large number of sRNAs await elucidation.

The current strategy for sRNA target discovery usually comprises bioinformatics prediction followed by experimental validation (3). For example, Richter *et al*. firstly applied the IntaRNA program to predict the targets of sRNA Yfr1 in *Prochlorococcus* MED4 (14,15); then, they chose six potential targets for validation, and finally found the two targets PMM1119 and PMM1121. Besides IntaRNA (15), other programs such as TargetRNA (16), TargetRNA2 (17), RNApredator (18), sRNATarget (19), sTarPicker (20) and the recently developed model CopraRNA (21) are available. However, the common shortcoming of these models is their high false positive rates. For example, the performances of the four models IntaRNA (15), TargetRNA2 (17) without considering ‘sRNA conservation and accessibility’, RNAPredator (18) and CopraRNA (21) were compared using a benchmark data set composed of 17 sRNA-target mRNA interactions. Five sRNAs were involved. The results indicated that among the top 35 predictions, IntaRNA, TargetRNA2, RNAPredator and CopraRNA detected 2, 3, 1 and 11 targets, respectively. Therefore, CopraRNA has the highest sensitivity ∼64.7%. However, the positive prediction value is only ∼6.3%. These results indicated that as many validated sRNA-target interactions as possible should be collected to optimize the related parameters in the models.

To provide support for sRNA functional studies such as developing sRNA target prediction models, we previously generated a database, sRNATarBase 2.0 (2), for sRNA targets verified by experiments. It contains 138 sRNA-target interactions and 252 non-interaction molecules. To date, the database has been applied in many aspects. For example, this database has been used to develop sRNA target prediction models, including sRNATarget (19), sTarPicker (20) and bistaRNA (22). Thébault employed part of the data set as a benchmark to evaluate prediction models, RNAup, IntaRNA and ssearch (23). Li incorporated the 138 sRNA-target interactions from sRNATarBase2.0 into another database BSRD (12). Our database also provided the data set for sequence and secondary structure analysis of sRNA target-binding regions (24–26). Additionally, the data have been applied to facilitating experimental studies of sRNA–mRNA interactions (27,28). As mentioned by Miyakoshi (29), in the era of systematic screening of sRNA functions, a curated sRNA target database, such as sRNATarBase, will be needed to identify obvious false positives among target activation patterns. However, many new targets have been discovered since the release of sRNATarBase 2.0. To provide a comprehensive and timely support to the sRNA research community, we recently updated the database. The new version contains 771 sRNA-target entries collected from 213 articles manually. A total of 492 and 316 validated interactions and binding regions, respectively, are involved. We also applied the models sRNATarget (19) and sTarPicker (20) to predict targets for each sRNA. Around 23 290 targets were predicted by sRNATarget, while 11 750 were obtained using sTarPicker. Additionally, we provided other new features such as NCBI sequence viewer for sRNA-target interaction (30), display of sRNA regulatory network (31), predicted targets-based GO and KEGG pathway annotations (32) and other functions. In comparison with the aforementioned databases, sRNATarBase 3.0 not only holds the largest number of sRNA-target interactions, but also provides the detailed information regarding sRNA-target binding regions and related mutation experiments. The new version will provide comprehensive support for bacterial sRNA functional studies.

## DATA COLLECTION

**Figure 1.**
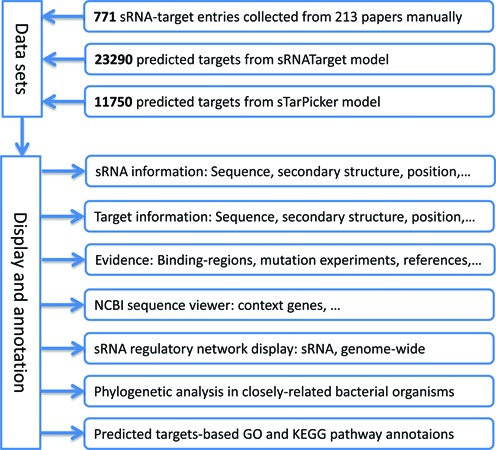
Flowchart of data collection and database construction. Firstly, 201 sRNAs were extracted from 771 sRNA-target entries, which were collected from 213 papers manually. Then, both sRNATarget and sTarPicker were applied to predict the targets of those sRNAs, and the number of predicted targets was 23 290 and 11 750, respectively. Finally, the database was developed using the targets above, and some tools for displaying and annotating sRNA-target interactions are provided, including sRNA information, target information, evidence supporting entries, NCBI sequence viewer, sRNA regulatory network, phylogenetic analysis, and predicted targets-based GO and KEGG pathway annotations.

To update the database, we first queried PubMed using keywords such as ‘bacterial sRNA function’ or ‘bacterial sRNA target’ or ‘bacterial small regulatory RNA target’, and found 4356 publications. Considering that sRNATarBase 2.0 took into consideration articles published before May 1, 2010, we mainly focused on those published between January 1, 2010 and June 1, 2015, i.e. 3124 papers (see Supplementary Table S1 for detailed information). Then, their abstracts were carefully reviewed, and 121 papers associated with sRNA-target interactions extracted. From these papers, information about sRNA-target entries was extracted. Combining the entries from sRNATarBase 2.0, we finally obtained 771 sRNA-target entries, including 492 with validated interactions and 279 with no reported interactions. For each interaction, relevant information was recorded as much as possible: sRNA sequences and genomic positions; target mRNA sequences and genomic positions; binding regions of sRNA–mRNA interactions and related mutation experiments; validation methods such as ‘Reporter assay’, ‘Mutation’, ‘Knock out’, ‘sRNA deletion’ and ‘Footprinting’. Additionally, we applied the latest version of NCBI genome sequences to update all entries, ensuring accuracy as much as possible. For each sRNA, we also provided predicted targets from sRNATarget (19) and sTarPicker (20). The detailed information regarding these entries is provided in Supplementary Table S2, which shows 752 sRNA–mRNA and 19 sRNA–protein entries distributed in 53 genomes. Among the 771 entries, 475 were validated RNA–mRNA interactions, while 17 sRNA–protein interactions were found. A total of 316 binding regions of sRNA–mRNA interactions were obtained. The flowchart of data collection and database construction is provided in Figure 1.

## DATABASE CONSTRUCTION AND ANNOTATION

**Figure 2.**
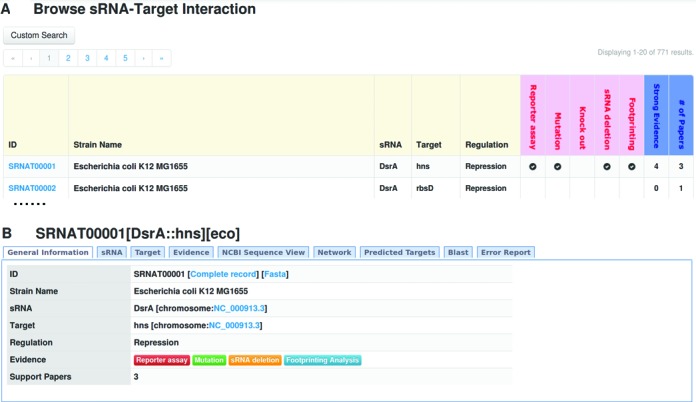
Overview of database entries. (**A**) The basic information for all entries is provided, including ID (‘SRNAT’ followed by a five-digit number), Strain name, sRNA name, Target name, Regulation type (Induction, Repression, Protein titration and No interaction) and Strong evidences (‘Reporter assay’, ‘Mutation’, ‘Knock out’, ‘sRNA deletion’ and ‘Footprinting’). (**B**) Detailed information for each entry is presented in tab pages named ‘General Information’, ‘sRNA’, ‘Target’, ‘Evidence’, ‘NCBI Sequence View’, ‘Network’, ‘Predicted targets’, ‘Blast’ and ‘Error report’.

When detailed information was obtained for each sRNA-target entry, related table files were constructed in CSV format, and imported into the MySql database. The Web interface for the database was rebuilt using the Yii framework (http://www.yiiframework.com) and PHP language, which is more friendly and intuitive than the previous version. Figure 2A provides an overview of database entries, in which each entry is depicted by its ID, strain name, sRNA name, target name, regulation type and experimental support. Here, five experimental strategies, i.e. ‘Reporter assay’, ‘Mutation’, ‘Knock out’, ‘sRNA deletion’ and ‘Footprinting’, were considered a strong support for sRNA-target interactions. Additionally, the detailed information was provided by hyperlink for each ID (Figure 2B). As shown in Figure 2B, each entry was annotated by the following tab pages: ‘General Information’, ‘sRNA’, ‘Target’, ‘Evidence’, ‘NCBI Sequence View’, ‘Network’, ‘Predicted targets’, ‘Blast’ and ‘Error report’. Since some tab pages have been described in the previous version, we only focus on new features, i.e. ‘NCBI Sequence View’, ‘Network’, ‘Predicted Targets’ and ‘Error report’.

## SEQUENCE VIEWER FOR sRNA-TARGET INTERACTIONS

**Figure 3.**
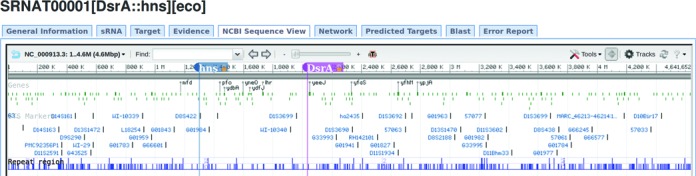
Display of DsrA-hns interaction using NCBI sequence viewer.

To view the genomic positions of sRNA-target mRNA interactions graphically, the NCBI sequence viewer was integrated into the server (30). For each entry, the viewer cannot only display the genomic positions of a sRNA and its target, but also provide information concerning the flanking genes. This component can also be used to annotate sRNA-target interactions. For example, blast comparison can be used to search the selected sequence against the NCBI nucleotide database. Figure 3 is a demonstration of DsrA-hns interaction, from which some flanking genes can be viewed.

## DISPLAY OF sRNA REGULATORY NETWORK

**Figure 4.**
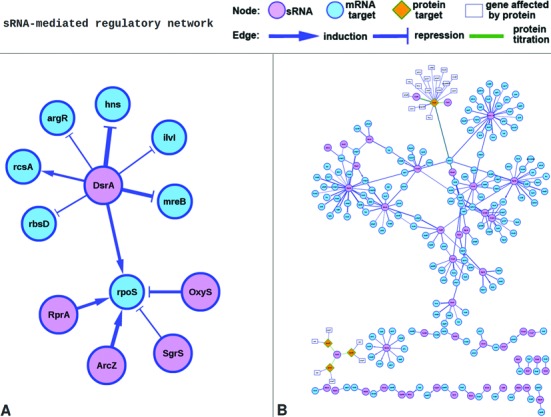
sRNA regulatory network. (**A**) The first-order regulatory network for the entry SRNAT00001 (DsrA-hns Interaction) is displayed, in which all validated targets (rpoS, argR, rbsD, hns, rcsA, mreB and ilvl) of the sRNA (DsrA) and sRNAs (DsrA, RprA, SgrS, OxyS and ArcZ) regulating the target (rpoS) are included. (**B**) genome-wide sRNA regulatory networks for the *E.coli* K12 MG1655 are displayed.

To improve the understanding of sRNA-target interactions, the CytoscapeWeb component was integrated into the server to display sRNA-target regulatory networks (31). For each sRNA-target interaction, all validated sRNA targets and sRNAs regulating the target will be displayed in the network. Figure 4A is an example of a regulatory network derived from the entry SRNAT00001 (DsrA-hns interaction), in which all DsrA targets (rpoS, argR, rbsD, hns, rcsA, mreB and ilvl) and sRNAs (DsrA, RprA, SgrS, OxyS and ArcZ) regulating a given target (i.e rpoS) are displayed. As shown in Figure 4A, the expression of the targets argR, rbsD, hns, mreB and ilvl can be repressed by DsrA. Additionally, DsrA can induce rpoS and rcsA expression. The target rpoS is regulated by five sRNAs (DsrA, RprA, SgrS, OxyS and ArcZ), among which DsrA, RprA and ArcZ are inducers, and the remaining two sRNAs (SgrS and OxyS) repressors. Here, we only provide first-order regulatory network. The whole regulatory network for a strain can be obtained through the menu bar ‘regulatory network’. Figure 4B represents the regulatory network integrating all sRNA-target interactions from *E.coli K12* MG1655.

## PREDICTION OF sRNA TARGETS

For most sRNAs in sRNATarBase 3.0, although some targets have been experimentally verified, the number of validated targets is still low. On average, 2.31 (391/169) targets were validated per sRNA (169 and 391 sRNAs and targets, respectively, are involved in the 492 interactions). These low target numbers may only represent a portion of sRNAs’ diverse regulatory functions. To study sRNA functions using GO or pathway annotations (32), both sRNATarget and sTarPicker were applied to predict targets for each sRNA included in the database. Then, the predicted targets were submitted to the DAVID webserver for sRNA functional annotation. Table 1 is an example of GO annotation for sRNA RybB in *E.coli*. According to previous reports (33,34), RybB is a sRNA that regulates outer membrane protein expression. As shown in Table 1, in addition to GO terms associated with membrane, GO terms associated with other functions such as ‘carbohydrate catabolic process’ and ‘polyol metabolic process’ were obtained.

**Table 1.** Both sRNATarget and sTarPicker were applied to predicting the targets of sRNA RybB in *E. coli*, and 163 and 59 targets were found, respectively. Then, these predicted targets were separately submitted to the David annotation webpage, and main GO annotation results were displayed

No.	sRNATarget			sTarPicker		
	Term	*P-* value	Benjamini	Term	*P-*value	Benjamini
1	peptidoglycan-based cell wall	5.6E-9	2.1E-7	peptidoglycan-based cell wall	2.3E-3	5.7E-2
2	cell wall	7.9E-9	1.5E-7	cell wall	2.6E-3	3.2E-2
3	organelle envelope	1.0E-8	1.3E-7	carbohydrate catabolic process	4.9E-3	6.6E-1
4	organelle inner membrane	1.0E-8	1.3E-7	transferase activity, transferring aldehyde or ketonic groups	7.4E-3	5.8E-1
5	organelle membrane	1.6E-8	1.4E-7	organelle envelope	1.6E-2	1.3E-1
6	carbohydrate catabolic process	3.6E-7	1.4E-4	organelle inner membrane	1.6E-2	1.3E-1
7	alditol metabolic process	8.7E-7	1.6E-4	organelle membrane	1.8E-2	1.1E-1
8	polyol metabolic process	9.5E-7	1.2E-4	pentose metabolic process	2.7E-2	9.5E-1
9	glycerol metabolic process	1.9E-5	1.8E-3	O antigen metabolic process	3.3E-2	9.1E-1
10	dicarboxylic acid metabolic process	8.7E-5	6.5E-3	O antigen biosynthetic process	3.3E-2	9.1E-1
11	plasma membrane	1.3E-4	9.9E-4	pentose-phosphate shunt, non-oxidative branch	3.3E-2	9.1E-1
12	external encapsulating structure	2.0E-4	1.2E-3	external encapsulating structure	4.0E-2	1.8E-1

## OTHER FUNCTIONS

In sRNATarBase 3.0, besides the aforementioned new features, we kept some elements of the previous version, including blast comparison, phylogenetic analysis and links to sRNA target prediction. Additionally, we provided other functions such as error report page, from which users can report errors of database entries so that data can be timely corrected.

## CONCLUSION AND FUTURE DEVELOPMENTS

In summary, we have updated the sRNA target database sRNATarBase. The new version holds 771 entries collected from 213 articles manually. The validated sRNA-target interactions and binding regions reached 492 and 316, respectively. In comparison with related databases such as RegulonDB (9), BSRD (12) and NPInter v2.0 (13), sRNATarBase 3.0 not only provides the largest number of bacterial interactions, but also includes 279 non-interactions, as well as detailed information about 316 sRNA-target mRNA binding regions and related mutation experiments. Data update along with new features, including NCBI sequence viewer (30), sRNA regulatory network (31), predicted target-based GO and pathway annotations (32) and other functions, make this database a useful resource for developing prediction models for binding regions of sRNA–mRNA interactions, and related sRNA functional annotations.

In the future, emphasis should be given to the following three points. First, the database will be updated regularly to provide support to the sRNA research community. Second, the benchmark data set from the database can be applied to develop new prediction models for sRNA targets or evaluate the performance of existing sRNA target prediction models. For example, data from the previous version were used to develop sRNATarget (19) and sTarPicker (20). At present, despite the developed models, the false positive rate is still high. Therefore, model parameters should be optimized using a large data set. This is also the purpose to develop and update the database. Third, the gene expression data set should be integrated into the database so that sRNA–mRNA interactions can be investigated from the perspective of gene expression. We believe that sequence-based sRNA target prediction only provides a potential interaction, while the study of gene expression-based sRNA-target interactions present dynamic explanations of sRNA-target relationship. Finally, this database will provide a comprehensive support for bacterial sRNA functional studies.

## AVAILABILITY

The sRNATarBase 3.0 and related information is available at http://ccb1.bmi.ac.cn/srnatarbase/.
